# Pattools‑implemented methylation vector analysis reveals aberrant subtype‑specific methylation in lung cancer across tissue and plasma cfDNA

**DOI:** 10.1002/ctm2.70812

**Published:** 2026-08-28

**Authors:** Zehua Dong, Yifeng Luo, Tingting Hu, Yihang Cheng, Qiaoling Ren, Li Xu, Yuan Tan, Wei Li, Yaoxiang Sun, Mingzhi Chen, Zhonghua Shen, Bin Zhang, Youhuang Bai, Yue Tao, Zhihong Cao, Deqiang Sun

**Affiliations:** ^1^ Department of Cardiology of the Second Affiliated Hospital, School of Medicine Zhejiang University Hangzhou China; ^2^ State Key Laboratory of Transvascular Implantation Devices Zhejiang University Hangzhou China; ^3^ Heart Regeneration and Repair Key Laboratory of Zhejiang Province Hangzhou China; ^4^ Department of Radiology The Affiliated Yixing Hospital of Jiangsu University Yixing China; ^5^ Department of Research and Development Zhejiang Gaomei Genomics Hangzhou China; ^6^ Information and Communication Engineering Ningbo University Ningbo China; ^7^ Key Laboratory of Biological Targeting Diagnosis, Therapy and Rehabilitation of Guangdong Higher Education Institutes The Fifth Affiliated Hospital of Guangzhou Medical University Guangzhou China; ^8^ Department of Clinical Laboratory The Affiliated Yixing Hospital of Jiangsu University Yixing China; ^9^ Department of Thoracic and Cardiovascular Surgery The Affiliated Yixing Hospital of Jiangsu University Yixing China; ^10^ Department of Cardiovascular Surgery of the Second Affiliated Hospital, School of Medicine Zhejiang University Hangzhou China; ^11^ College of Food Science and Engineering Ningbo University Ningbo China; ^12^ School of Traditional Chinese Medicine Shanghai University of Traditional Chinese Medicine Shanghai China

**Keywords:** cell of origin, cfDNA, epigenetic biomarker, lung cancer subtypes, methylation vector

## Abstract

**Introduction:**

Lung cancer is characterised by high mortality and encompasses various subtypes with markedly different treatment outcomes. While individual subtypes have been studied, comprehensive genome‐wide and fine‐scale DNA methylation analyses across subtypes remain unexplored. This study aims to identify true subtype‐specific aberrant methylation by developing a novel method that eliminates cell‐origin and immune confounding, moving beyond traditional approaches.

**Methods:**

We assembled an in‐house cohort of 115 tissue samples across five groups (CTL, LUAD, LUSC, LCC, SCLC) with detailed clinicopathological annotations. Matched plasma cfDNA samples (*n* = 24; 9 LUAD, 7 LUSC, 8 healthy) were collected for translational assessment. We developed an MV analysis method implemented in the open‐source toolkit pattools to differentiate cell‐specific fragments in bulk BS‐seq data and identify subtype‐specific methylation vector regions (SMVRs).

**Results:**

Genome‐wide methylation profiling revealed that subtype‐specific signals originate from distinct cell types—alveolar epithelium in LUAD, head and neck epithelium in LUSC, fibroblasts in LCC, and endocrine cells in SCLC—with additional immune infiltration influences. Using pattools‐based MV analysis, we identified 500 key SMVRs per subtype. In matched plasma cfDNA, these tissue‐derived SMVRs demonstrated promising discriminatory power for LUAD (AUC = 0.98, 95% CI: 0.95 to 1.00) and LUSC (AUC = 0.88, 95% CI: 0.79 to 0.97) in binary classification, and AUCs of 0.95 (95% CI: 0.90 to 1.00), 0.75 (95% CI: 0.62 to 0.88), and 0.63 (95% CI: 0.48 to 0.78) for LUAD, LUSC and healthy samples, respectively, in multi‐class classification.

**Conclusion:**

The MV analysis in pattools effectively uncovers subtype‐specific aberrant methylation signals, offering potential for precise diagnosis and subtyping in tissue and liquid biopsy. Independent validation in larger cohorts is required before clinical translation.

## Graphical abstract

1. 115 tissue samples across five lung cancer subtypes and matched plasma cfDNA were profiled by WGBS.

2. Pattools implements methylation vector (MV) clustering to isolate tumour‐specific fragments from immune and cell‐origin signals in bulk BS‐seq.

3. MV analysis identified 500 subtype‐specific SMVRs per subtype, with promising plasma classification performance (LUAD AUC = 0.98; LUSC AUC = 0.88), pending independent validation.

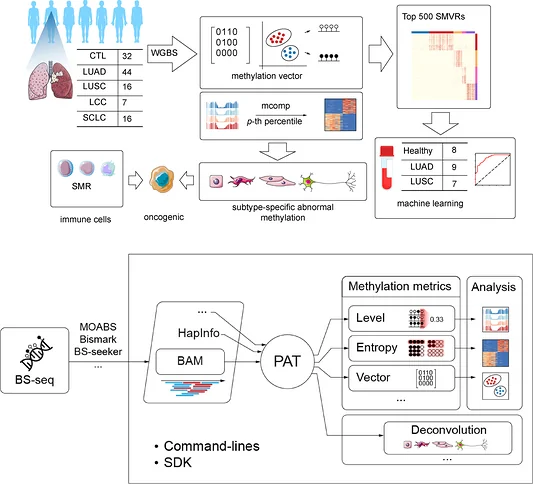

## 1 INTRODUCTION

Lung cancer is characterised by a high incidence and mortality rate,^1^ posing a significant threat to human health. Investigating the distinct histological subtypes of lung cancer is critical for advancing detection and treatment strategies. Lung cancer is primarily classified into two major histological subtypes: non‐small cell lung cancer (NSCLC) and small cell lung cancer (SCLC), accounting for approximately 85% and 15% of all lung cancer cases, respectively. NSCLC can be further subdivided into lung adenocarcinoma (LUAD, approximately 40%), lung squamous cell carcinoma (LUSC, approximately 25%), and lung large cell carcinoma (LCC, < 10%), among others.^2^ LCC includes large cell neuroendocrine carcinoma (LCNEC),^3^ basaloid carcinoma, and other types. Despite advances in treatment, accurate identification of lung cancer subtypes remains a key challenge, as each subtype requires distinct therapeutic approaches and exhibits varying prognoses.^4^, ^5^, ^6^ Accurate identification of lung cancer subtypes remains a key challenge, and understanding the cell of origin is vital for classification and treatment. Current evidence indicates that lung cancer subtypes are determined by both the cell of origin and driver mutations.^7^ However, the precise epigenetic mechanisms underlying subtype specification remain incompletely understood.

DNA methylation is a critical epigenetic regulatory mechanism involved in tumourigenesis.^8^ With the advent of whole‐genome bisulphite sequencing (WGBS), the epigenetic landscape of cancer can now be analysed at single‐nucleotide resolution. While methylation level^9^ is the most commonly used metric and is compatible with array‐based data, it has inherent limitations: it represents an average across mixed cell populations and cannot distinguish methylation patterns from different cellular sources within bulk tissues. In recent years, Mendelian randomisation (MR) has advanced considerably, particularly in cardiovascular diseases^10^ and cancer research.^11^ To complement tissue‐based analyses, we employed summary‐data‐based Mendelian randomisation (SMR) analysis^12^ using publicly available GWAS and mQTL data to investigate abnormal methylation signals in the blood of patients with different lung cancer subtypes. This dual‐tissue approach provides a more comprehensive view of methylation abnormalities in lung cancer.

Another important measure is methylation haplotypes (mHap), which reflect the methylation status of consecutive CpG sites on a single DNA fragment.^13^ Various metrics have been developed based on mHap, including methylation entropy,^14^ the proportion of discordant reads,^15^ cellular heterogeneity‐adjusted clonal methylation,^16^ methylation concurrence ratio,^17^ and methylated haplotype load.^18^ However, all current metrics describing methylation status are scalar values that lose information about the spatial arrangement of methylation within individual DNA fragments. In bulk BS‐seq, DNA fragments from various cells are combined, making it difficult to differentiate methylation signals from individual cells. While single‐cell sequencing technologies can overcome this limitation, they remain costly and difficult to apply to large sample sizes.

To address these limitations, we propose a novel vector‐based indicator—methylation vectors (MVs)—to investigate the DNA methylation status of consecutive CpGs in lung cancer. Unlike scalar metrics, MVs preserve the spatial methylation pattern within individual DNA fragments, enabling the differentiation of cell‐specific fragments within bulk BS‐seq data. By using MV analysis implemented in pattools (https://github.com/hcyvan/pattools), we aimed to uncover the true subtype‐specific abnormal methylation signals in lung cancer, eliminating confounding from cell‐of‐origin and immune cell infiltration.

## 2 MATERIAL AND METHODS

### 2.1 Patients and sample acquisition

Clinical data and samples were collected from 115 patients with pulmonary conditions at Yixing Hospital, Jiangsu University, including 32 CTL (non‐malignant pulmonary tissues: Hamartoma, *n* = 2; Inflammation, *n* = 18; Normal lung, *n* = 4; Fibrosis, *n* = 5; Tuberculosis, *n* = 3), 7 LCC, 44 LUAD, 16 LUSC, and 16 SCLC cases. Detailed demographic information (age, sex, smoking history, and histopathological diagnosis) for each CTL subgroup is provided in Table S1, and sequencing quality metrics stratified by CTL subgroup are summarised in Table S2. Diagnosis was confirmed by two pathologists through independent review of Hematoxylin and Eosin slides.

### 2.2 WGBS library preparation and data analysis

Genomic DNA was extracted using the DNeasy Blood & Tissue Kit (Qiagen) for library prep. The DNA, along with 1% unmethylated lambda DNA (Promega), was sonicated to generate 100–700 bp fragments, then converted with the EZ DNA Methylation‐Gold Kit (Zymo Research). Sequencing was done on the Illumina HiSeq6000. WGBS data was processed with MOABS (v1.3.9.6).

### 2.3 The *p_t_* algorithm for filtering subtype‐specific DMCs

In this context, the group being analysed for subtype‐specific DMCs is referred to as the target group; in contrast, the remaining four groups are designated as the background group. The *p_t_* of the target group and the (100 – *p*)th percentile (*p_b_*) of the background group are calculated, respectively.

$$\begin{array}{ccc} p_{low} & = & \left\{\begin{array}{cc} p_{t}, & \text{for hypo DMCs}\\ p_{b}, & \text{for hyper DMCs} \end{array}\right.,\;p_{high}\\ & = & \left\{\begin{array}{cc} p_{b}, & \text{for hypo DMCs}\\ p_{t}, & \text{for hyper DMCs} \end{array}\right. \end{array}$$

$$P=\max_{p_{low}\leq p_{high}}p_{t}$$

If a *p_t_* meeting these conditions is found, the DMC is termed the *p_t_* DMC. This algorithm is implemented in methytools (v0.9.1) and can be accessed via the code repository (epiLungCancer) (v1.0) associated with this article.

### 2.4 Connecting subtype‐specific DMCs to subtype‐specific DMRs

Multiple DMCs are merged into a single DMR if two conditions are satisfied: (1) the distance between adjacent DMCs is no more than 200 bp and (2) the DMR contains at least three DMCs. The detailed steps for this process are implemented in methytools (mcomppost dmc2dmr).

### 2.5 MV analysis

We represent methylated CpGs as 1 and unmethylated CpGs as 0, utilising a 0–1 array to indicate the methylation status of consecutive CpGs within the genome. This vector, composed of *n* binary values, is referred to as a MV.

The MV analysis described in this study involves three primary steps:

(1) Vectorisation: A sliding window containing *w* consecutive CpGs is used to traverse the genome, advancing one CpG site at a time (window cutting). The continuous CpGs within each window are converted into 0–1 vectors, and any CpG motifs that do not meet the length *w* are discarded. This step was implemented using the pattools mv‐vectorisation command. For each sample, a sliding window of length *w* CpGs is applied with a step size of 1 CpG site, resulting in a series of overlapping windows where adjacent windows share *w* – 1 CpGs. Within each window, methylated CpGs are represented as 1 and unmethylated CpGs as 0, forming a 0–1 vector. Vectors with dimensions less than *w* are discarded.
(2) Clustering: The MVs from all samples are merged based on their position within the window, with each MV labelled according to its corresponding sample and sample group. The MVs in each window are clustered using a novel algorithm developed in this study, termed multiple repeats and equal spacing clustering (MRESC). This step was implemented using the pattools mv‐clustering command. Merge MVs from all samples of CTL, LUAD, LUSC, LCC, and SCLC, and label each MV according to its sample and sample group of origin within each window. Apply MRESC algorithm to cluster the MVs in each window. Alternatively, existing clustering algorithms such as DBSCAN,^19^ HDBSCAN,^20^ or KMeans^21^ may be used in place of MRESC for this step.
(3) Separating: This step was implemented using the pattools mv‐separating command. The samples and sample groups from which the MVs in each cluster originate are identified. Two key metrics are then calculated for the MVs in each cluster:
(a) The proportion of MVs from a specific group relative to the total number of MVs (SMVP).
(b) The proportion of samples from the specific group relative to the total number of samples in that group (SSP).

In a given window of MVs across samples from *n* groups, *m* clusters are obtained after clustering.

$$\begin{array}{c} C_{k}\in \left\{C_{1},C_{2},\text{…}C_{m}\right\},\;k\in \left(1,2,\text{…},m\right),\\ G_{j}\in \left\{G_{1},G_{2},\text{…}G_{n}\right\},\;j\in \left(1,2,\text{…},n\right). \end{array}$$

For the *j*th group, the formula for calculating SMVP in the *k*th cluster is:

$$\mathrm{SMV}P_{jk}=\frac{MV_{k}\left(G_{j}\right)}{\sum_{i=1}^{n}MV_{k}\left(G_{i}\right)},$$

where *MV_k_* represents the number of MVs in cluster *k*, and *MV_k_* (*G_j_*) represents the number of MVs from group *G_j_* within cluster *k*.

Similarly, the formula for calculating SSP for the *j*th group in the *k*th cluster is:

$$\mathrm{SS}P_{jk}=\frac{S_{jk}\left(G_{j}\right)}{\sum_{i=1}^{n}S_{jk}\left(G_{i}\right)},$$

where *S_jk_* is the number of samples from group *j* in cluster *k*, and *S_jk_* (*G_j_*) is the number of samples from group *G_j_*.

The algorithm has been implemented in pattools (https://github.com/hcyvan/pattools) (v1.2.0).

### 2.6 Definition of SMVCs and SMVRs

A cluster is considered a subtype‑specific methylation vector cluster (SMVC) if it satisfies two conditions: (1) SMVP*_jk_* = 0, meaning that all MVs in the cluster originate exclusively from the target group; and (2) SSP*_jk_* ≥ 0.2, meaning that at least 20% of samples within the target group contribute MVs to this cluster (primary screening threshold). For final SMVC identification after immune reference filtering (using the Loyfer et al. methylation atlas to remove immune‑derived signals), we apply a more stringent threshold of SMVP = 0.95 (≥95% of MVs in the cluster originate from the target group) and SSP > 0.2 (present in >20% of target samples). This two‑step strategy balances discovery sensitivity (primary screening) with reporting specificity (final filtering).

Adjacent SMVCs are merged into subtype‑specific methylation vector regions (SMVRs) if (1) the distance between adjacent SMVCs ≤ 200 bp and (2) the merged region contains at least three SMVCs. SMVRs were ranked by the number of CpGs they contain, and the top 500 hypomethylated SMVRs per subtype were selected as key SMVRs (Table S10).

A step‐by‐step reproducible example of the complete MV analysis pipeline, from PAT file to SMVR identification, is provided in the Software and Code as a worked example. The example demonstrates the execution of three core pattools commands—pattools mv‐vectorization, pattools mv‐clustering, and pattools mv‐separating—with input file formats, command‐line parameters, intermediate outputs, and the final SMVR call, ensuring full reproducibility of the workflow.

### 2.7 Classification of hypomethylated and hypermethylated SMVCs

SMVCs are classified based on their distance to the all‑zero MV (representing complete unmethylation). For each SMVC, we calculate:

*d*1: the Manhattan distance between the centre of the SMVC cluster and the all‑zero MV
*d*0: the distance between the cluster furthest from the all‑zero MV and the all‑zero MV

If *d*1 < *d*0, the SMVC is classified as hypomethylated (the cluster centre is closer to complete unmethylation than to any other cluster, indicating a predominance of unmethylated fragments). If *d*1 > *d*0, it is classified as hypermethylated (the cluster centre is closer to complete methylation, indicating a predominance of methylated fragments).

### 2.8 Threshold sensitivity analysis

To assess the robustness of our threshold selection, we evaluated the number of SMVCs identified at SMVP = 0.90, 0.95, and 1.00 with SSP = 0.2. The results are summarised in Table S17. While the absolute number of SMVCs varied across thresholds, the relative ranking of SMVRs across subtypes remained stable (SCLC > LUSC > LCC > LUAD > CTL), and the top SMVRs were consistently recovered across all thresholds, confirming the robustness of our key findings. Based on these analyses, we selected SMVP = 0.95 and SSP = 0.2 as the reporting threshold, as it provides an optimal balance between specificity (≥95% of MVs originating from the target group) and sensitivity (present in ≥20% of target samples).

### 2.9 MRESC

In comparison to conventional clustering methods, the clustering of MVs exhibits 2 distinct characteristics: (1) A limited number of MVs types exist within a fixed window, with a significant number of these vectors recurring frequently. These identical MVs represent the same methylation motif. In a window containing *w* CpGs, there are 2*w* possible methylation motifs. Each motif may be observed multiple times. (2) Methylation motifs are distributed equidistantly, forming a grid‐like pattern. For example, the manhattan distance between the MVs (0, 0, 0, 0) and (1, 0, 0, 0) is 1, and similarly, the distance between (0, 0, 0, 0) and (0, 1, 0, 0) is also 1.

(1) In the representation of MVs, methylated CpGs are denoted by 1, while unmethylated CpGs are denoted by 0. In the representation of methylation motifs, ‘C’ represents methylated CpGs, and ‘T’ represents unmethylated CpGs. The distances between two distinct motifs are calculated and then sorted from smallest to largest. The distance between a given motif and itself is marked as 0. The distance between two adjacent moitfs is marked as 1. The distance between two moitfs separated by one interval is marked as 2, and so forth. In a window containing *w* CpGs, each methylation motif is adjacent to *w* neighbouring motifs, with a manhattan distance of 1.
(2) Here is the pseudo code of MRESC (Algorithm 1). There are several key functions in this algorithm.

**countVectorsInEachMotif**: Count the number of methylation vectors representing each distinct methylation motif.

**findNeighborsIndex**: Find the neighbouring motifs of a given motif

**findNeighborMax**: Identify the maximum MVs counts among the neighbouring motifs of a given motif

**markCluster**: Assign a cluster label to each motif

**assignClusterLabel2Vectors**: Assign a cluster label to each MV

Algorithm 1 MRESC

**Table:** 

**Function doMRESC**(vectors, w)
counts ← **countVectorsInEachMotif**(vectors)
clusters ← [0] * w
**For** i ← 0 to w ‐ 1
**For** j in **findNeighborsIndex**(i)
**If** counts[i] < counts[j] then
Continue
**EndIf**
max nb ← findNeighborMax(j, counts)
**If** counts[i] ≥ max nb then
markCluster(i, j, clusters)
**EndIf**
**EndFor**
labels ← assignClusterLabel2Vectors(vectors, clusters)
**Return** labels

### 2.10 Genome reference

The genome reference hg38 was used for processing WGBS data and other analyses.

### 2.11 Statistical analysis

Statistical tests and significance levels (ns: not significant, *p < .05, **p < .01, ***p < .001, ****p < .0001) are indicated in the figures, figure legends, and tables. For sensitivity analyses comparing methylation levels between CTL and pooled lung cancer or individual subtypes, two‐tailed Wilcoxon rank‐sum tests were used for two‐group comparisons, and Kruskal–Wallis tests were used for multi‐group comparisons. Effect sizes were calculated as Cohen's d with 95% confidence intervals (CIs). To correct for multiple hypothesis testing, FDR‐adjusted *p* values were calculated using the Benjamini–Hochberg method. For CTL subgroup sensitivity analyses, samples were stratified by histopathological diagnosis (inflammation, pulmonary fibrosis, normal lung, tuberculosis, and hamartoma). For smoking‐stratified analyses, samples with unknown smoking history were excluded. All sensitivity analyses were performed using the ggstatsplot and effsize packages in R (v4.3).

For the benchmarking analysis, features were selected from the top 50 subtype‐specific regions per subtype. Random forest classifiers were trained using default parameters (ntree = 200) with fivefold cross‐validation and stratified sampling to prevent sample leakage between training and test sets (detailed in Table S12).

### 2.12 UXM fragment‐level deconvolution algorithm

UXM was calculated as the fraction of DNA fragments with all CpGs unmethylated within a given genomic region, following the definition of Loyfer et al. For each SMVR, the UXM value was computed as the number of fully unmethylated fragments divided by the total number of fragments, multiplied by 100.

### 2.13 Benchmarking

To benchmark SMVRs against DMRs, we performed fivefold cross‐validation using random forest classifiers, extracting features from the top 50 subtype‐specific regions per subtype. Performance was evaluated using AUC with 95% confidence intervals.

## 3 RESULTS

### 3.1 Genome‐wide DNA methylation profiles in lung cancer

**FIGURE 1:**
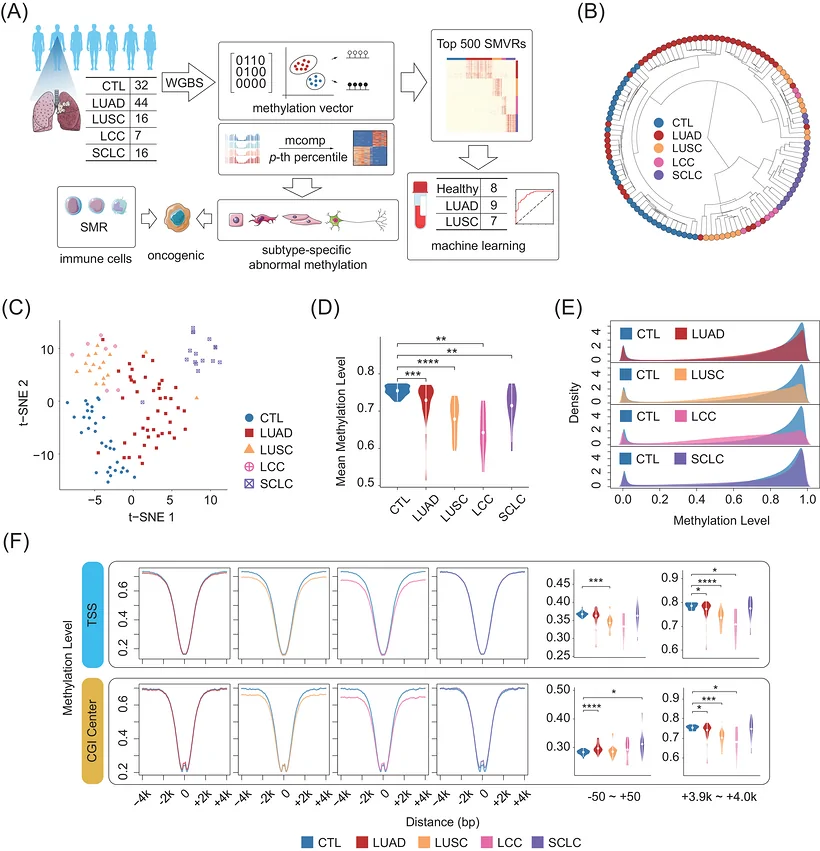
Experimental design and genome‐wide DNA methylation profiles across all types of lung cancer. (A) Overview of the experimental design. (B) Cluster analysis of methylation data from all samples. (C) t‐SNE analysis of methylation data from all samples.(D) Comparative analysis of mean DNA methylation level across the CTL, LUAD, LUSC, LCC, and SCLC groups. (E) Distribution of DNA methylation densities for LUAD, LUSC, LCC, and SCLC compared with CTL. (F) Methylation level analysis within a 4000 bp range upstream and downstream of the TSS (top) and CGIs (bottom) across the CTL, LUAD, LUSC, LCC, and SCLC groups. The violin plot on the right shows methylation levels for each sample group within the proximal 100 bp region (–50 to approximately +50) and the distal region (+3.9k to approximately +4.0k) from the TSS/CGI centre. CTL, control; LUAD, lung adenocarcinoma; LUSC, lung squamous cell carcinoma; LCC, lung large cell carcinoma; SCLC, small cell lung cancer; TSS, transcription start site; CGIs, CpG islands.

In this study, 115 subjects were recruited and categorised into five groups: control (CTL), LUAD, LUSC, LCC, and SCLC (Figure 1A; Table S1). Samples and data from the CTL and LUAD groups have been utilised and analysed in our previous study.^22^ A cohort representing the main subtypes of lung cancer was obtained by collecting new samples from patients with lung cancer (Table 1). Age did not serve as a determining factor for grouping; however, gender and smoking history exhibited varying proportions across the different groups. To minimise the potential influence of gender on the analysis, results from the sex chromosomes were excluded. Given that 37% of individuals in this cohort had an unknown smoking history and that smoking was not the primary focus of this study, smoking factors were not considered further.

**TABLE 1:** Summary of clinical information for subjects.

		CTL	LUAD	LUSC	LCC	SCLC	Total	*p* value	Effect size	95% CI	FDR‐adjusted *p* values
Gender											
	Male	20	18	16	6	15	75	.0000186^a^	Cramér's V = 0.46	(0.31 to 0.58)	.000056
	Female	12	26	0	1	1	40				
Age (years)											
	Median	57	64.5	67.5	65	60	62	.22^b^	*η* ^2^ = 0.04	(−2.1 to 8.7)	.22
	Average	58.16	63.41	64.31	61.29	58.67	61.29				
	Range	34 ‐ 78	45 ‐ 83	48 ‐ 78	47 ‐ 74	41‐80	34 ‐ 83				
Smoke history											
	Smoker	7	3	6	2	3	21	.000000986^a^	Cramér's V = 0.56	(0.42 to 0.67)	.000003
	Non‐smoker	18	31	1	0	1	51				
	Unknown	7	10	9	5	12	43				
Histopathology											
	Hamartoma	2									
	Inflammation	18									
	Normal lung	4									
	Pulmonary fibrosis	5									
	Tuberculosis	3									

Note: FDR‐adjusted p values were calculated using the Benjamini–Hochberg method across all tests performed in this table. Effect sizes: Cramér's V for Chi‐squared tests, η^2^ for Kruskal–Wallis test. 95% CIs were calculated using bootstrap resampling.

^a^Analysed by Chi‐squared test.

^b^Analysed by Kruskal–Wallis test.

All newly enrolled samples underwent WGBS experiments. Following quality control, WGBS data were obtained with a bisulphite conversion rate of 99.41% (95% confidence interval [CI]: 99.40% to 99.43%) and a CpG coverage depth of 25.0 (95% CI: 24.2 to 25.9) (Table S2). Samples with more than 10 CpG counts were selected for hierarchical clustering and t‐distributed stochastic neighbour embedding analysis (Figure 1B and C). Most samples within each group clustered together, with LUSC, LCC, and SCLC samples distinguishable from CTL samples. However, some LUAD samples were mixed with CTL and other cancer groups, which may be attributable to tumour heterogeneity.

Compared to the CTL group, all four cancer groups showed significantly reduced average methylation levels (Figure 1D). Methylation density in NSCLC, especially LUSC and LCC, differed from the CTL group, while SCLC resembled the CTL group (Figure 1E). Genome‐wide, all subtypes showed hypomethylation at transcription start sites (TSS) and CpG island (CGI) centres (Figure 1F). Near the TSS (–50 bp to +50 bp), only LCC showed significant methylation reduction. At CGI centres, LUAD and SCLC exhibited increased methylation. In regions further from the TSS and CGI (+3900 bp to +4000 bp), NSCLC showed reduced methylation, while SCLC had no significant change. These findings suggest that NSCLC exhibits widespread methylation changes near TSS and CGIs, while SCLC changes are mainly at CGI centres.

To assess the robustness of the observed methylation differences and to address potential confounding from the heterogeneous composition of the control group, we performed a series of sensitivity analyses. Global methylation levels were significantly lower in pooled lung cancer samples compared to CTL (Wilcoxon test *p <* .001, Figure S4A). Subtype‐specific comparisons confirmed that this hypomethylation was consistent across LUAD, LUSC, LCC, and SCLC (all FDR‐adjusted *p* < .001; Table S13). Stratification of CTL samples by histopathological diagnosis (inflammation, fibrosis, normal lung, tuberculosis, and hamartoma) revealed that all CTL subgroups clustered separately from cancer samples, with no substantial confounding from control group heterogeneity (Figure S4B; Table S14). Furthermore, the CTL versus cancer methylation difference remained significant when stratified by smoking status (Table S15).

### 3.2 Subtype‐specific CpG sites based on methylation level in lung cancer

**FIGURE 2:**
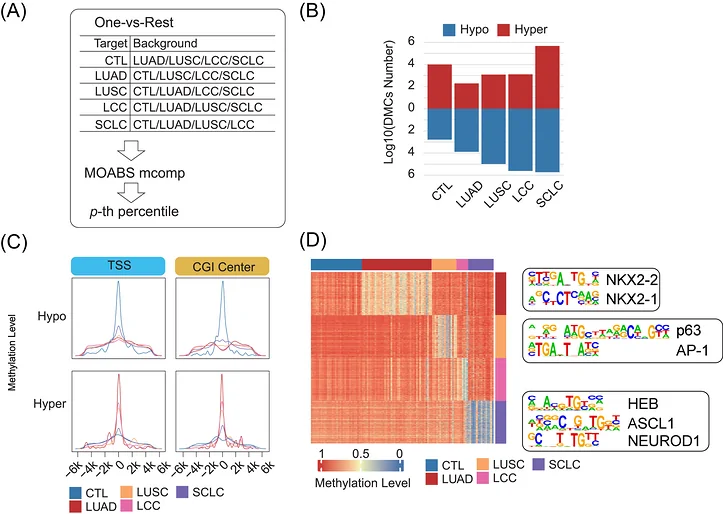
Subtype‐specific DMCs and DMRs in lung cancer. (A) Flowchart illustrating the process of identifying subtype‐specific DMCs. (B) The number of subtype‐specific DMCs identified, with blue representing hypomethylated DMCs and red representing hypermethylated DMCs. (C) Density plots showing the distribution of subtype‐specific DMCs near the TSS (left) and within CGIs (right). The different colours in the curve represent different lung cancer subtypes. (D) Heatmap depicting the hypomethylated subtype‐specific DMRs and the TFs enriched in these regions. DMCs, differentially methylated CpGs; TSS, transcription start site; CGIs, CpG islands; DMRs, differentially methylated regions.

To investigate subtype‐specific methylation sites based on methylation level in lung cancer, a one‐vs‐rest strategy was employed for the differential analysis of methylation levels across all CpGs in the genome (Figure 2A). The comparison between the CTL and cancer groups aimed to identify the common features of various lung cancer subtypes. Inspired by a previous study,^23^ the *p_t_* algorithm and related software tools were developed to filter the differentially methylated CpGs (DMCs) obtained in mcomp,^9^ resulting in DMCs that exhibited enhanced subtype specificity and were more suitable as biomarkers. By filtering out CpGs with a *p_t_* less than 80, four groups of subtype‐specific DMCs, and one group of subtype‐shared DMCs were identified. The numbers of LUAD‐, LUSC‐, LCC‐, and SCLC‐specific DMCs were 7771, 97 307, 407 399, and 966 325, respectively; in contrast, the number of subtype‐shared (CTL‐specific) DMCs was 10 466 (Figure 2B). Unlike NSCLC, SCLC contained a large number of hypermethylated DMCs, comparable to the number of hypomethylated DMCs (Table S3). This observation explains why SCLC exhibits a methylation density distribution more similar to that of the CTL group despite having more DMCs than NSCLC (Figure 1E and F). The distribution of these DMCs in the genome was then examined, revealing that the density of hypomethylated and hypermethylated DMCs in NSCLC compared with CTL was higher at the TSS and the centres of CGI (Figure 2C).

To explore the biological functions of these DMCs, adjacent DMCs were connected to form differentially methylated regions (DMRs). Consequently, 241, 644, 6784, 19 524, and 77 888 DMRs were identified for CTL, LUAD, LUSC, LCC, and SCLC, respectively. Notably, the majority of these DMRs were hypomethylated (Table S4). The DMRs were categorised based on the number of CpGs, and the top 500 DMRs within these hypomethylated regions were selected as key subtype‐specific DMRs for lung cancer (Table S5). Using the HOMER (v4.11) tool,^24^ transcription factor (TF) binding motifs were enriched in these key subtype‐specific DMRs (Figure 2D; Table S6). NKX2‐1 was enriched in hypomethylated LUAD‐specific DMRs. These findings align with those from our previous study, and confirming the expression patterns of NKX2‐1 in LUAD.^25^ As a marker of basal cells,^26^ p63 was enriched in LUSC, suggesting that LUSC may originate from these cells. AP‐1 was also enriched in LUSC and has been demonstrated to play a significant role in various types of cancer. Furthermore, in SCLC‐specific DMRs, ASCL1 and NEUROD1—TFs that play critical roles in SCLC^27^ and the nervous system^28^—were enriched.

### 3.3 The joint impact of abnormal methylation in origin cells and immune cells on lung cancer

**FIGURE 3:**
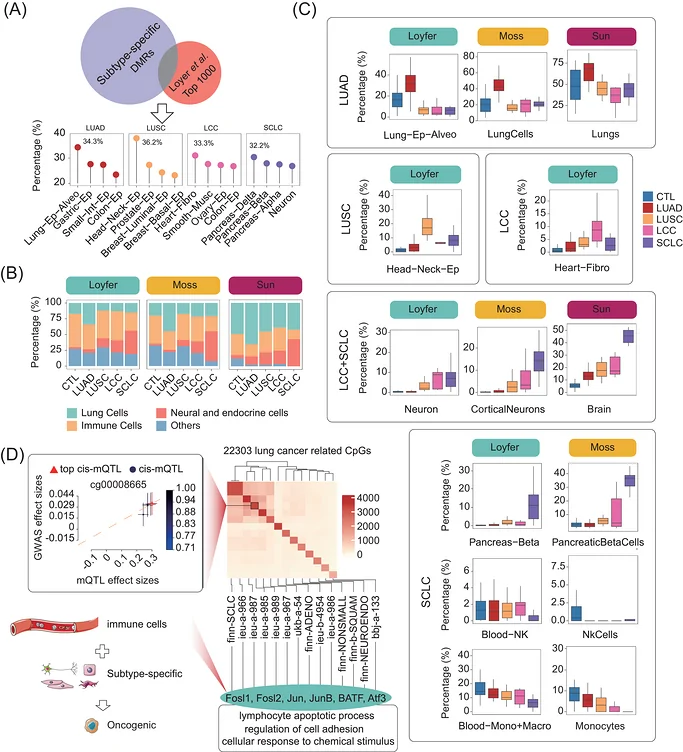
Abnormal methylation signals in lung cancer tissues and blood across different subtypes. (A) Proportions of various cell type‐specific markers in the overlapping regions between lung cancer subtype‐specific DMRs and the top 1000 hypomethylated loci from human cell type‐specific data. (B) Cell proportions within each lung cancer tissue group as determined by the three deconvolution algorithms: Loyfer et al. (left), Moss et al. (middle), and Sun et al. (right). (C) Proportion of certain cell types in different lung cancer subtypes. Boxplots within rounded boxes indicate the dominant or disadvantaged cell types for each lung cancer subtype. Each boxplot is labelled according to the deconvolution algorithm used. (D) SMR analysis across various datasets for each lung cancer subtype. The heatmap shows the number of lung cancer‐related CpGs identified in blood samples from each dataset. Below the heatmap, the enrichment of common transcription factors and biological processes, identified through HOMER and GREAT analyses, is presented for each dataset. DMRs, differentially methylated regions; SMR, summary‐data‐based Mendelian randomisation; GREAT, Genomic Regions Enrichment of Annotations Tool.

To investigate the sources of subtype‐specific methylation signals in each lung cancer subtype, a comparison was made between specific methylation sites in each subtype and the human DNA methylation atlas.^23^ Among the subtype‐specific DMRs, several cell‐type‐specific loci were identified. The composition of these shared regions varied significantly with changes in lung cancer subtypes (Figure 3A). In LUAD, 34.3% of the overlapping regions originated from the lung alveolar epithelium. In LUSC, 36.2% of the overlapping areas were derived from head and neck epithelium, with the remaining areas sourced from the epithelium of various organs, including lung bronchial epithelium. In LCC, the overlapping regions predominantly originated from fibroblasts and epithelium. In SCLC, 32.2% of the overlapping areas were traced back to endocrine cells, with most of the remaining areas coming from neurons and epithelium.

The cell type‐specific methylation signals observed in lung cancer subtype‐specific sites suggest an increase in the proportion of certain cell types in lung cancer tissues, likely due to the disordered proliferation of these cells following malignancy. To verify the cellular proportions across different lung cancer subtypes, three distinct methods^23^, ^29^, ^30^ were employed to deconvolute the lung cancer group. To minimise the interference from the presence of numerous cell types, specific cell and tissue types were selected for analysis in lung cancer (Table S7, Supplementary Material). The results obtained using different algorithms were consistent (Figures 3B and S1A and B). LUAD was predominantly composed of lung cells, with the algorithm by Loyfer et al.^23^ indicating that these cells were mainly lung alveolar epithelium. This finding aligns with results from other studies and corroborates the cellular origin of LUAD.^31^, ^32^, ^33^, ^34^ In SCLC, a significant number of methylation signals from neural and endocrine cells were observed, suggesting that SCLC cells may participate in the biological processes of both the nervous and endocrine systems. This indirectly supports the hypothesis that SCLC originates from neuroendocrine cells.^35^

Differences in methylation signals from specific cell types were observed across various lung cancer tissue types (Figure 3C). All three deconvolution algorithms indicate that, in LUAD tissue, the signal from lung alveolar epithelium was higher than in other groups. Since not all algorithms support all cell types, some cell type‐specific methylation signals were validated only in certain algorithms. For instance, a prominent signal from head and neck epithelium was found in LUSC, a strong signal from fibroblasts was observed in LCC, and a high signal from endocrine cells was detected in SCLC. The higher methylation signatures of certain cell types may be shared across multiple lung cancer subtypes. For example, nervous system‐related cell signals in LCC and SCLC were significantly higher than in other lung cancer subtypes, likely due to the neuroendocrine origin of some LCC cases.^3^ Additionally, the methylation signal of specific cell types was lower in certain lung cancer subtypes; in SCLC, the methylation signals of NK cells and monocytes were significantly lower compared to other subtypes.

To assess the influence of additional factors, such as immune environment, on lung cancer, SMR analysis was conducted across various lung cancer subtypes using publicly available data. The dataset encompasses nearly 100 000 patient samples and over 20 million single nucleotide polymorphisms, distinguishing between multiple lung cancer subtypes, including squamous cell carcinoma, adenocarcinoma, SCLC, and neuroendocrine lung cancer (Table S8). After obtaining the SMR analysis results, a filtering process was applied using the criteria SMR *p* < .05 and HEIDI *p* > .05 to ensure the robustness and reliability of the findings. Ultimately, a total of 22 303 CpG sites associated with lung cancer were identified across 14 datasets (Figures 3D and S2A). The SMR results obtained from various lung cancer subtypes were consistently replicated across the datasets. HOMER analysis was performed on the identified CpGs, leading to the enrichment of several TFs associated with lung cancer across different datasets. Notably, TFs from the AP‐1 family were prevalent in most datasets (Figures 3D and S2B). GREAT (v4.0.4) analysis indicated that the CpGs associated with lung cancer were likely involved in key biological processes, including lymphocyte apoptosis, regulation of cell adhesion, and cellular responses to chemical stimuli (Figures 3D and S2C). These findings suggest that abnormal methylation signals in the cells of origin for different lung cancer subtypes, combined with the dysregulated biological functions of immune cells, contribute to the development of lung cancer.

### 3.4 Utilisation of MVs analysis in lung cancer

**FIGURE 4:**
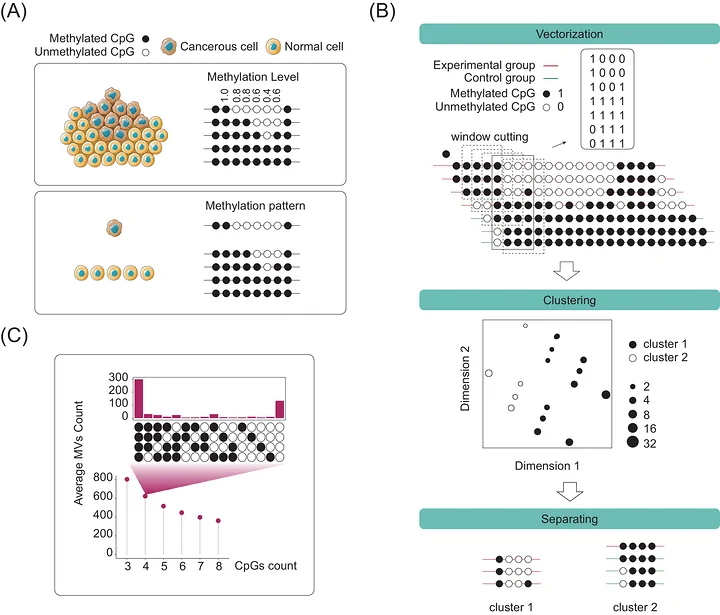
Methylation vector analysis in lung cancer. (A) Schematic representation of bulk BS‐seq data. The methylation level indicates the ratio of methylated cytosines to total cytosines at a CpG site (top). Distinct methylation patterns are exhibited by fragments from different cells (bottom). (B) Workflow for methylation vector analysis. (C) Number of MVs across different window sizes in lung cancer. BS‐seq, bisulphite sequencing; MVs, methylation vectors.

The methylation level is defined as the ratio of methylated CpGs at a single site to the total number of CpGs (Figure 4A). However, this metric is not suitable for assessing the methylation status across multiple CpG sites. Additionally, in bulk BS‐seq, this measure is susceptible to interference from background signals, such as methylation signals from immune cells. To isolate unique DNA fragments from different subtypes of lung cancer tissues and investigate their distinct methylation patterns, we propose a vector methylation metric along with related algorithms. Methylated CpGs are denoted by 1, and unmethylated CpGs by 0, allowing continuous CpGs to be represented as a vector composed of 0s and 1s. This vector is referred to as a MV. MV analysis consists of three main steps: vectorisation, clustering, and separation (Figure 4B). In the vectorisation step, BS‐seq data are segmented into overlapping windows, and the methylation motifs within each window are converted into vectors. Subsequently, the set of vectors within each window is clustered in the Cartesian space defined by the window. Finally, the cluster unique to the experimental group is isolated, and the vectors within this cluster are identified as those specific to the experimental group. All code related to MVs analysis was initially implemented within the pattools developed by our team.

The MVs analysis was conducted on 27 852 739 CpG sites located on 22 pairs of autosomes in lung cancer samples. Attempts were made to perform the analysis using windows containing three to eight CpG sites. After excluding windows that did not contain MVs, it was observed that the number of MVs within a window gradually decreased as the MV dimension increased. Following the merging of 115 samples analysed in this study, an average of 802 MVs were identified in windows containing three CpG sites; in contrast, an average of 361 MVs were found in windows containing eight CpG sites (Figure 4C). Within a window, MVs consisting entirely of 1s or entirely of 0s were significantly more prevalent than other MV configurations. This suggests that complete methylation and complete unmethylation represent the more favourable methylation status for a given DNA sequence. Additionally, the coverage of available windows (those containing at least one MV) also declined as the window length increased (Table S9).

A preliminary screening of subtype‐specific MVs (SMVs) was conducted. Following clustering and separation steps, screening thresholds for group‐specific MV proportion (smvp) and group‐specific sample proportion (SSP) were set at 0.90 and 0.30, respectively. It was observed that as the window length increased, the number of subtype‐specific MV clusters (SMVCs) initially increased and then decreased. The window‐cutting scheme utilising windows of five to seven CpG sites yielded the highest number of SMVCs across various lung cancer subtypes (Figure S3). Vectorisation schemes with varying window lengths were compared, leading to the selection of a window containing four CpGs for subsequent analysis. At a coverage of 76.79%, each window included an average of 623.3 MVs, corresponding to an average of 5.4 MVs per sample.

### 3.5 Subtype‐specific MV regions in lung cancer reveal the authentic abnormal methylation signals

**FIGURE 5:**
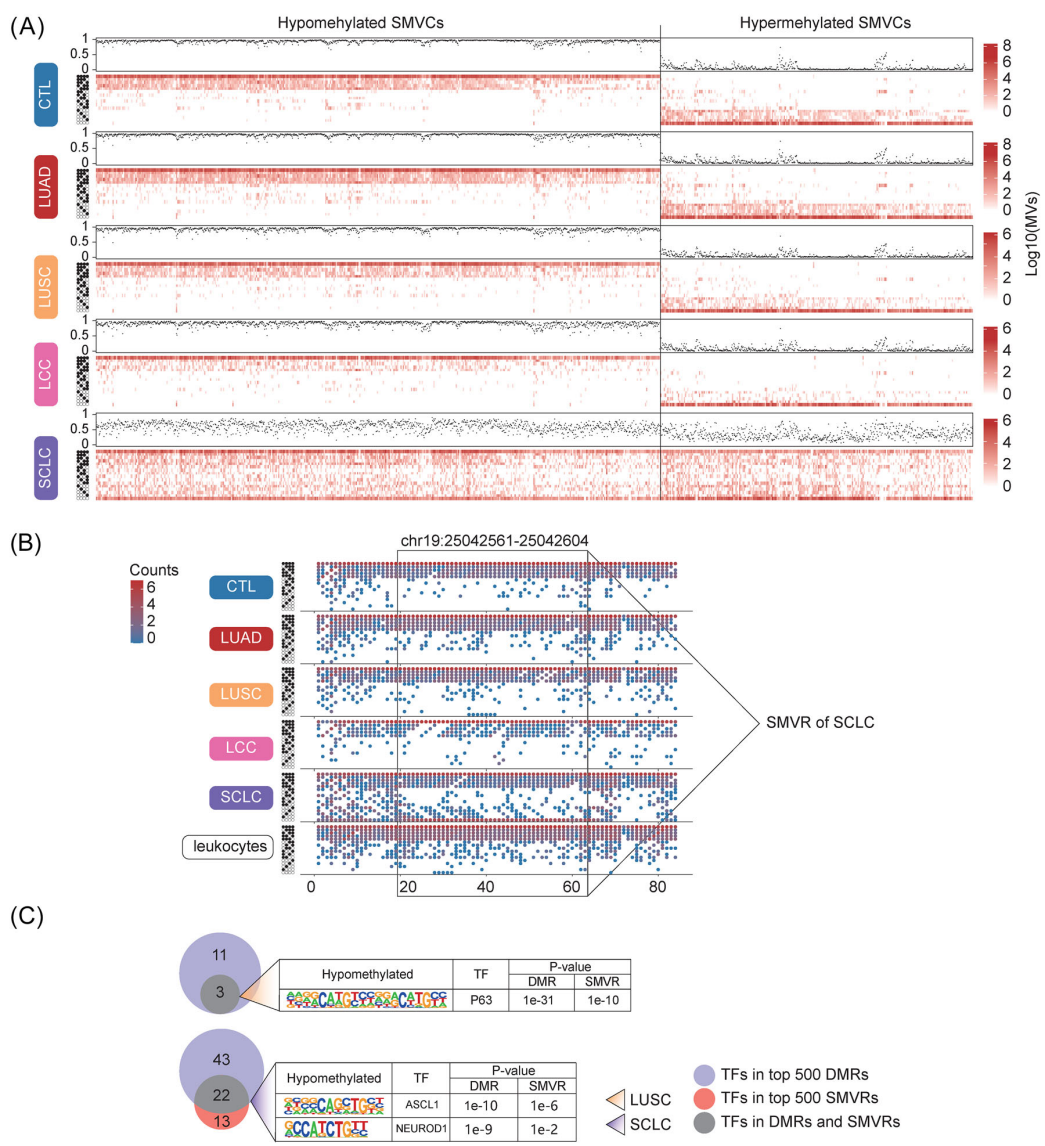
SMVCs and SMVRs in lung cancer. (A) Distribution of MVs in the SCLC‐specific SMVC window across lung cancer subtypes. Black circles on the left denote methylated CpGs; in contrast, white circles represent unmethylated CpGs. The chessboard diagram illustrates 16 distinct MVs. (B) Heatmap depicting one SMVR in SCLC (chr19:25042561‐25042604). Each row in the heatmap corresponds to a group, with annotations on the left representing CTL, LUAD, LUSC, LCC, SCLC and leukocytes (GSE186458). The dots in the heatmap represent the quantity of MVs at each location. (C) TFs enriched in SMVC windows and subtype‐specific DMRs. SMVCs, subtype‐specific methylation vector clusters; SMVRs, subtype‐specific methylation vector regions; MVs, methylation vectors; SCLC, small cell lung cancer; CTL, control; LUAD, lung adenocarcinoma; LUSC, lung squamous cell carcinoma; LCC, large cell carcinoma; DMRs, differentially methylated regions; TFs, transcription factor.

To eliminate the influence of immune cells on lung cancer methylation, immune cell methylation data from Loyfer et al.^23^ were utilised as a reference background. MVs analysis was then conducted in conjunction with the methylation data from our cohort of 115 subjects. By setting the conditions to SMVP = 0.95 and SSP > 0.2, a total of 2396, 10 703, 8678, and 11 597 SMVCs were identified in LUAD, LUSC, LCC, and SCLC, respectively. Based on the distance between the cluster centre and the completely unmethylated MVs, these SMVCs were further categorised into hypomethylated and hypermethylated SMVCs. In regions containing hypomethylated SMVCs, completely unmethylated contiguous CpGs were found exclusively in certain cancer subtypes. Conversely, in regions with hypermethylated SMVCs, fully methylated contiguous CpGs were observed only in specific cancer subtypes (Figure 5A). Adjacent SMVCs were merged to form subtype‐specific MV regions (SMVRs), which were subsequently analysed. It was observed that the number of hypo SMVRs exceeded that of hyper SMVRs, aligning with the patterns identified using methylation level‐based indicators (DMRs). Furthermore, SMVRs were ranked based on the number of CpGs they contained, and the top 500 hypo SMVRs for each lung cancer subtype were selected as key SMVRs in lung cancer (Table S10). For example, one of the SMVRs in SCLC is located within the genomic region at chr19:25042561‐25042604 (Figure 5B). In this region, long continuous unmethylated CpGs were identified in SCLC samples. This particular methylation pattern was not observed in other lung cancer subtypes or in leukocytes.

HOMER analysis of these SMVRs revealed an enrichment of TF binding sites (Table S11). Some of these TF binding sites also appeared in subtype‐specific DMRs, likely because SMVRs can induce changes in methylation levels. In LUSC, an enrichment of three TFs—P63, P53, and P73—was identified in both LUSC‐specific DMRs and SMVRs. Similarly, 22 TFs, including ASCL1 and NEUROD1, were enriched in both LUSC and SCLC‐specific DMRs and SMVRs (Figure 5C). Unlike DMRs, which are based on methylation levels, SMVRs derived from MVs can isolate specific methylation fragments from complex background signals. For instance, it was found that ASCL1 and NEUROD1 could bind to the genomic region at chr19:25042561‐25042604, potentially due to the presence of four consecutive unmethylated CpGs within this region.

### 3.6 SMVRs as a biomarker for lung cancer subtypes in tissue and plasma

**FIGURE 6:**
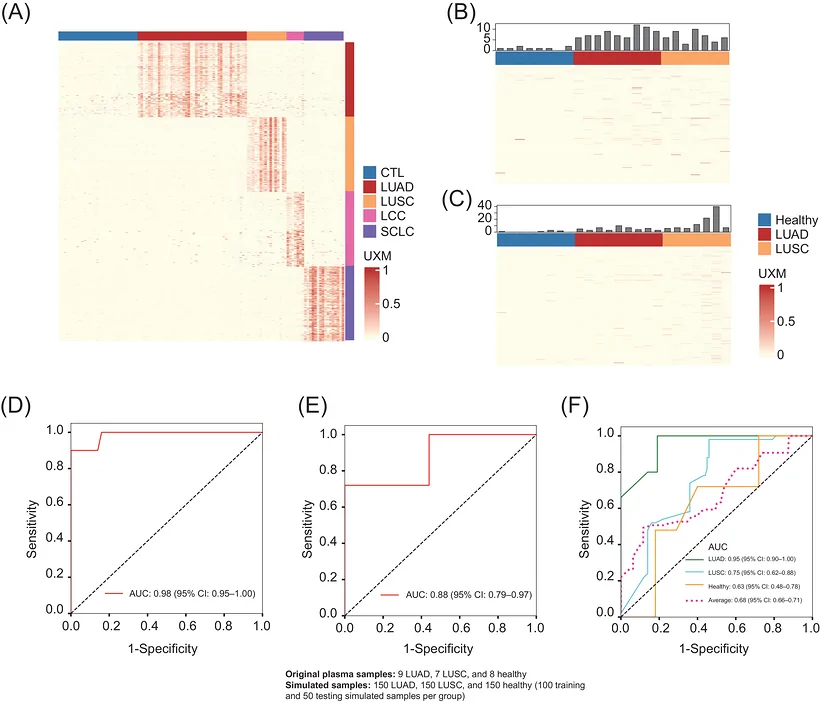
UXM of SMVRs for differentiating lung cancer subtypes in tissue and plasma. (A) UXM of SMVRs in tissue samples from different lung cancer subtypes. (B, C) UXM of LUAD‐specific (B) and LUSC‐specific (C) SMVRs in plasma samples. (D, E) ROC curves of the binary classification random forest model for LUAD vs. healthy plasma (D) and LUSC vs. healthy plasma (E). (F) ROC curves of the multi‐classification random forest model for LUAD, LUSC and healthy plasma. SMVRs, subtype‐specific methylation vector regions; LUAD, lung adenocarcinoma; LUSC, lung squamous cell carcinoma.

To investigate whether the key SMVRs can be used to identify lung cancer and its subtypes, we analysed them in both lung cancer tissues and plasma samples. The UXM,^23^ a fragment‑level metric defined by Loyfer et al. that quantifies the proportion of completely unmethylated DNA fragments within a given genomic region, was calculated as an indicator for the evaluation of hypo SMVRs. Because hypomethylated SMVRs are defined by the predominance of completely unmethylated contiguous CpGs in a subtype‑specific manner, and UXM directly captures the proportion of such fully unmethylated fragments at the fragment level, this metric is particularly suitable for validating and quantifying the hypomethylation status of SMVRs across different sample groups. UXM in the SMVRs of 115 tissue samples were extracted using pattools. It was found that the UXM of SMVRs differed significantly among lung cancer tissue samples of various subtypes (Figure 6A). The SMVRs of each subtype exhibited high UXM values, suggesting the presence of hypomethylated fragments in these regions, which aligns with the results derived from MVs analysis. This finding indicates that the UXM of SMVRs can serve as a reliable metric to differentiate between various subtypes of lung cancer tissue.

To investigate whether DNA fragments in SMVRs of lung cancer tissues can be released into the blood, plasma samples were collected from 9 LUAD, 7 LUSC, and 8 healthy subjects. Using cfDNA WGBS data from these samples, an analysis was conducted to determine whether SMVRs in plasma samples contained hypomethylated fragments originating from tumour tissues. It was observed that only a small proportion of the top 500 hypo SMVRs in LUAD and LUSC plasma contained hypomethylated fragments. Within the SMVRs of LUAD, the plasma of LUAD and LUSC subjects were found to contain more hypomethylated fragments compared to the plasma of healthy subjects (Figure 6B). Similarly, within the SMVRs of LUSC, the plasma of LUSC patients exhibited a higher abundance of hypomethylated fragments than those of LUAD subjects and healthy subjects (Figure 6C). To more accurately assess the performance of SMVRs in distinguishing different lung cancer subtypes, plasma from LUAD, LUSC, and healthy subjects was mixed in pairs at varying proportions. A total of 100 simulated samples were generated as the train dataset and 50 as the test dataset (Table S12). A random forest binary classification model was applied to distinguish between LUAD and healthy plasma, as well as LUSC and healthy plasma. The results demonstrated that the AUCs of the LUAD and LUSC identification models reached 0.98 (Figure 6D) and 0.88 (Figure 6E), respectively, indicating that SMVRs can effectively differentiate between plasma from lung cancer and healthy subjects. Additionally, a random forest multi‐classification model was employed to distinguish among LUAD, LUSC, and healthy plasma. The AUCs for identifying LUAD, LUSC, and healthy samples were 0.95 (95% CI: 0.90 to 1.00), 0.75 (95% CI: 0.62 to 0.88), and 0.63 (95% CI: 0.48 to 0.78), respectively, with an average AUC of 0.68 (95% CI: 0.66 to 0.71) (Figure 6F). These findings suggest that SMVRs show promise in the multi‐classification model; however, the identification of healthy plasma (AUC = 0.63) requires further improvement. This limitation is attributable to the small number of real plasma samples (9 LUAD, 7 LUSC, 8 healthy) and the simulation‐based nature of the training and test datasets. Additionally, the absence of LCC and SCLC plasma samples precludes evaluation of the model's performance in these subtypes. Independent validation in a larger real‐world cfDNA cohort is therefore essential before clinical translation.

## 4 DISCUSSION

Lung cancer remains a leading cause of morbidity and mortality worldwide. The physiological and genetic characteristics of different lung cancer subtypes vary considerably, necessitating distinct therapeutic approaches. Early classification and diagnosis of lung cancer subtypes are crucial for reducing cancer mortality.^1^ Although studies have been conducted on individual lung cancer subtypes,^36^, ^37^, ^38^ comprehensive genome‐wide and fine‐scale methylation analyses that integrate all subtypes remain limited. This study collected data from over 100 patients with lung cancer or pulmonary diseases, including non‐cancerous conditions and the four major types of lung cancer: LUAD, LUSC, LCC, and SCLC (Figure 1A). Compared to single‐subtype analyses, a comprehensive approach allows for the identification of subtype‐specific abnormal methylation signals, which can be leveraged for lung cancer classification and identification. Our benchmarking analysis further confirmed the incremental value of SMVRs over DMR‐based biomarkers across all four subtypes, with AUC improvements of 0.037–0.153 (Table S16). This supports the notion that fragment‐level MV clustering captures methylation signals that are partially diluted in bulk methylation averages.

Abnormal methylation signals were identified in both lung cancer tissues and blood. Hypomethylated, subtype‐specific DMRs in LUAD were enriched for NKX2‐1, which displays high expression patterns in LUAD.^25^ In hypomethylated LUSC‐specific DMRs, p63, a basal cell marker TF,^26^ was enriched, supporting the hypothesis that LUSC originates from basal cells. Additionally, ASCL1 and NEUROD1 were enriched in the hypomethylated DMRs of SCLC, consistent with studies that have used their expression levels for SCLC classification.^27^ These TFs are highly expressed in their respective cell types, likely occupying DNA binding regions and contributing to abnormal methylation patterns. Methylation abnormalities in lung cancer subtypes are closely linked to the tumour's cell of origin. In LUAD, methylation signals were identified from epithelial tissues such as the colon, small intestine, alveoli, and kidney. In LUSC, methylation signals were detected from epithelial cells of the head, neck, and bronchial tissues. In LCC, methylation signals were associated with nervous system cells and cardiac fibroblasts; in contrast, in SCLC, signals were identified from the nervous system and pancreatic islet cells. Traditional methylation level‐based analysis methods struggle to distinguish cell origin‐related and immune‐related signals from subtype‐specific abnormal methylation signals.

To overcome the limitations of traditional analysis methods, the concept of the MV was introduced. Through vectorisation, clustering, and separation, subtype‐specific SMVCs were identified for each lung cancer subtype. Unlike algorithms based on methylation levels, the MV‐based algorithm requires lower sequencing depth and tumour purity. During clustering, all samples are merged, allowing the lower sequencing depth and tumour purity of individual samples to be compensated by other samples within the group. Moreover, the MV algorithm is compatible with BS‐seq data from various sample types and sequencing strategies. By merging SMVCs, top 500 key SMVRs for each lung cancer subtype were identified. Our benchmarking analysis demonstrated that SMVRs achieve superior classification performance compared to conventional DMR‐based biomarkers, with AUC improvements of 0.08–0.15 across subtypes, supporting the incremental value of the MV approach. These SMVRs reflect the true abnormal methylation signals in lung cancer and can serve as biomarkers for distinguishing between different lung cancer subtypes in both tissues and plasma.

Compared to conventional diagnostic approaches for lung cancer subtyping—which rely on histopathological examination, immunohistochemistry, and molecular testing—the SMVR‐based approach offers several potential advantages. First, as a methylation‐based biomarker, SMVRs can be assessed from cfDNA in plasma, enabling non‐invasive liquid biopsy‐based subtyping that avoids the risks associated with tissue biopsy. Second, the MV algorithm requires lower sequencing depth and tumour purity than conventional methylation level‐based methods, potentially making it more applicable to clinical samples with limited tumour content, such as early‐stage lesions or samples with low tumour cellularity. Third, by eliminating immune and cell‐origin confounding, SMVRs provide more specific subtype signals than traditional DMR‐based biomarkers. However, we acknowledge that these advantages remain to be validated in prospective clinical cohorts, and current diagnostic gold standards (histopathology and IHC) remain essential for clinical decision‐making. The SMVR approach should be viewed as a complementary tool that may eventually assist, but not replace, existing diagnostic workflows.

Emerging evidence suggests that alterations in TGF‐β signalling may influence immune exclusion, antigen presentation, T‐cell infiltration, and PD‐L1 expression in lung cancer.^39^, ^40^, ^41^, ^42^ While our methylation data reveal subtype‐specific epigenetic alterations, we acknowledge that the current study primarily supports association rather than causality. Nevertheless, the convergence of our SMVR findings with known TGF‐β‐related pathways in specific subtypes (particularly LUSC and SCLC) suggests potential mechanistic links that warrant future investigation. The integration of methylation biomarkers with established immunotherapy predictors such as PD‐L1 expression, TMB, and immune microenvironment features represents a promising direction for future research and is supported by recent studies demonstrating the role of methylation‐altered networks in shaping cancer hallmarks and immune pathway interplay patterns in lung cancer.^43^

Recent methodological advances in MR/SMR reproducibility, such as the MendelR package, provide valuable frameworks for future causal inference studies integrating methylation and genetic data.^44^ Additionally, novel methylation‐based prognostic models have been developed for lung adenocarcinoma,^45^ and DNA methylation biomarkers in both tissue and plasma have shown promise for early diagnosis of non‐small cell lung cancer.^46^ The convergence of our SMVR findings with known TGF‐β‐related pathways, together with recent evidence on TGFBR3 involvement in lung cancer development,^47^ suggests potential mechanistic links that warrant future investigation. Moreover, DNA methylation has been shown to cooperate with genomic alterations during non‐small cell lung cancer evolution, highlighting the need for methods that can resolve methylation heterogeneity at the fragment level.^48^ These observations further underscore the incremental value of our MV framework over existing methylation biomarkers and immune‐microenvironment studies.

However, several aspects of this study require further investigation. The research on MVs introduced here needs expansion. Due to the limited read length of second‐generation sequencing, mHap can only be analysed within a shorter spatial range. To address this limitation, future work will incorporate scBS‐seq and third‐generation sequencing. Additionally, to further validate the relevance of SMVRs in liquid biopsies, independent validation in large, real‐world cfDNA cohorts is essential before clinical application. We are currently collecting a larger independent cfDNA cohort (*n* > 100) for prospective validation. Despite these ongoing efforts, this study provides a comprehensive and detailed analysis of DNA methylation abnormalities across various lung cancer subtypes, offering a genome‐wide and fine‐scale epigenetic landscape for each subtype. A novel methylation vector‐based analysis method has been proposed, which enhances the ability to analyse tumour carcinogenic factors and enables the identification of more reliable biomarkers.

Several limitations of this study should be acknowledged. First, the control group includes various non‐malignant pulmonary conditions rather than solely healthy tissues; while sensitivity analyses demonstrated that these conditions do not confound the cancer‐specific signals, this remains a consideration for interpretation. Second, the cfDNA validation cohort is small (9 LUAD, 7 LUSC, 8 healthy) and lacks LCC and SCLC plasma samples; the training and testing of random forest models were performed on simulated mixtures derived from these limited samples, and the multi‐class AUC for healthy plasma (0.63) suggests that discrimination between healthy and cancer plasma requires further improvement. Independent validation in large, real‐world cfDNA cohorts is therefore essential before clinical application. Third, functional experiments to validate the biological roles of SMVRs were not performed. Finally, stage, treatment background, and mutation burden data were not available for all samples, limiting our ability to perform stratified analyses. The influence of tumour purity and sequencing depth on SMVR stability was not systematically evaluated; however, the MV algorithm is designed to require lower sequencing depth and tumour purity than conventional methylation‐level methods, which may mitigate these concerns.

## 5 CONCLUSION

In summary, this study introduces the methylation vector as a novel conceptual framework and pattools as its practical implementation, enabling a more nuanced analysis of DNA methylation data. By adopting a vector‑based approach, our method effectively reduces confounding interference from non‑tumour cells, uncovering subtype‑specific methylation landscapes. Applied to a cohort of 115 tissue samples across five lung cancer subtypes, this methodology identified 500 key SMVRs per subtype. Preliminary validation in matched plasma cfDNA demonstrated promising discriminatory power, underscoring their potential as biomarkers for non‑invasive diagnosis and subtyping. However, given the limited sample size of the cfDNA cohort, these findings should be interpreted as proof‑of‑concept, and independent validation in larger cohorts is required before clinical translation. These findings highlight the advantage of the methylation vector algorithm over traditional scalar methods and support its further application in liquid biopsies.

## AUTHOR CONTRIBUTIONS

ZD conceived the study, curated data, developed methodology, performed validation, and wrote the original draft. YL contributed to methodology and validation. TH participated in data curation, investigation, and formal analysis. YC handled data curation and software development. QR developed software. LX provided resources and participated in investigation. YT provided resources. WL provided resources. YS participated in investigation. MC participated in investigation. ZS provided resources. BZ supervised the research, administered the project, reviewed and edited the manuscript, and acquired funding. YB supervised the research, administered the project, and reviewed & edited the manuscript. YT supervised the research, and administered the project. ZC supervised the research, and reviewed & edited the manuscript. DS conceived the study, acquired funding, administered the project, supervised research, and reviewed & edited the manuscript. All authors read and approved the final manuscript.

## CONFLICT OF INTEREST STATEMENT

The authors declare that they have no known competing financial interests or personal relationships that could have appeared to influence the work reported in this paper.

Human DNA methylation atlas of normal cell types (GEO: GSE186458): this dataset, generated by Loyfer et al. and published in Nature (2023), comprises deep WGBS data of 39 cell types sorted from 205 healthy tissue samples. Replicates of the same cell type are > 99.5% identical, demonstrating the robustness of cell identity programs. Sequencing was performed on the Illumina NovaSeq 6000 platform. This atlas was used to trace the cell‐of‐origin of subtype‐specific methylation signals and to provide reference methylation data for immune cell deconvolution. Additional public GWAS summary datasets used for SMR analysis are listed in Table S8 with full accession details, sample sizes, and quality control parameters.

All custom scripts and software tools developed in this study are accessible through the GitHub repositories epiLungCancer (https://github.com/hcyvan/epiLungCancer) and pattools (https://github.com/hcyvan/pattools). Specific versions and commit hashes used in this study are as follows: epiLungCancer v1.0 (commit: ed78268cebc43a4279d9d28b3ec441f86a260009), pattools v1.2.0 (commit: 2a07820d6aa0b1c2f375c7cf82c125c3013e5edc), and methytools v0.9.1 (commit: 1e0890c0b65f6119cc8d312dcb1e086e9e8cd2b4). Key command lines for the core analysis steps—including tissue deconvolution:

 pattools deconv ‐m sun ‐g hg38 ∖

‐c /path/to/references/hg38/CpG.bed.gz ∖

‐p sample.pat.gz ‐o sample.sun.tsv ∖

–include Lungs Heart Brain T‐cells B‐cells Neutrophils

 and the MV analysis pipeline—are provided in the Software and Code, along with example input and output files. A minimal working example demonstrating the complete pipeline from PAT file to SMVR identification is also included in the Supplementary Material. All processed intermediate results have been deposited at CNGB‐NGDC (PRJCA024580) and are accessible for full reproducibility.

## ETHICS STATEMENT

All experiments involving animals were conducted according to the ethical policies and procedures approved by the ethics committee of the Affiliated Yixing Hospital of Jiangsu University, China (Approval no. ER‐2021‐040‌). This document confirms that the research involving human tissue samples and data was conducted in accordance with the ethical standards of the Declaration of Helsinki.

## SOFTWARE AND CODE

This document provides a comprehensive description of the custom scripts and software environment used for the methylation vector analysis.

## PLAGIARISM CHECK

The similarity report for the manuscript is generated using iThenticate. The overall similarity index was 6%, and all sources of matching text have been appropriately cited or are within the scope of common terminology.

## Supporting information



Supporting Information

Supporting Information

Supporting Information

Supporting Information

Supporting Information

## Data Availability

The raw BAM files generated in this study have been deposited at the CNGB‐NGDC under accession number PRJCA024580. Other processed data, including raw FASTQ files, processed methylation calls, PAT files, clinical metadata, and cfDNA data, are available upon request to the corresponding author.
