# Supplementary material for: Pattools‑implemented methylation vector analysis reveals aberrant subtype‑specific methylation in lung cancer across tissue and plasma cfDNA
Source: Clin Transl Med. 2026 Aug 28;16(9):e70812. doi: 10.1002/ctm2.70812 (PMC13522992; doi:10.1002/ctm2.70812)
Supplement: Supplementary file 1 [file CTM2-16-e70812-s001.pdf]

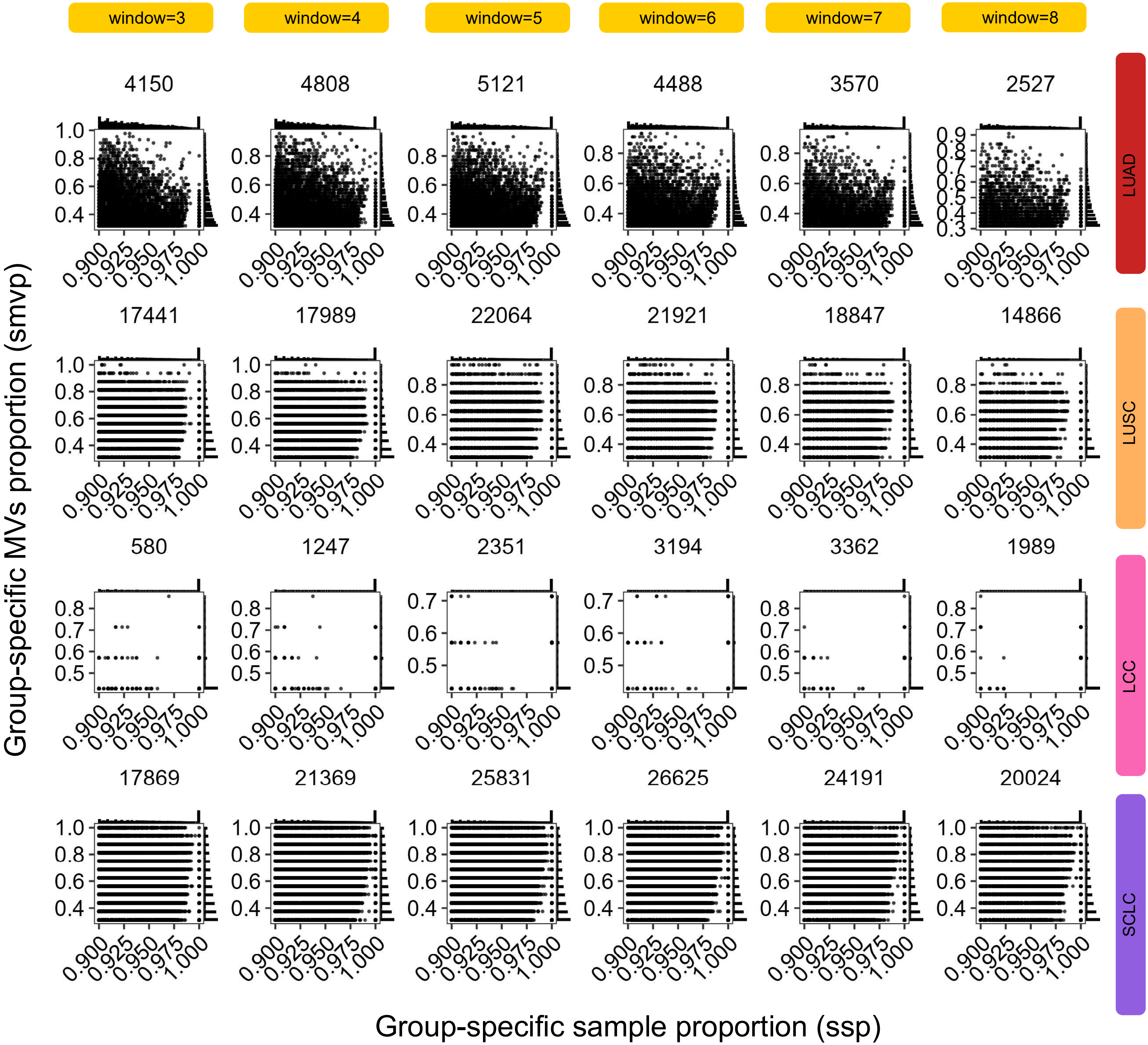
window=3
window=4
window=5
window=6
window=7
window=8
LUAD
LUSC
LCC
SCLC
4150
4808
5121
4488
3570
2527
17441
17989
22064
21921
18847
14866
580
1247
2351
3194
3362
1989
17869
21369
25831
26625
24191
20024
Group-specific MVs proportion (smvp)
Group-specific sample proportion (ssp)
