# Supplementary material for: Pattools‑implemented methylation vector analysis reveals aberrant subtype‑specific methylation in lung cancer across tissue and plasma cfDNA
Source: Clin Transl Med. 2026 Aug 28;16(9):e70812. doi: 10.1002/ctm2.70812 (PMC13522992; doi:10.1002/ctm2.70812)
Supplement: Supplementary file 2 [file CTM2-16-e70812-s002.pdf]

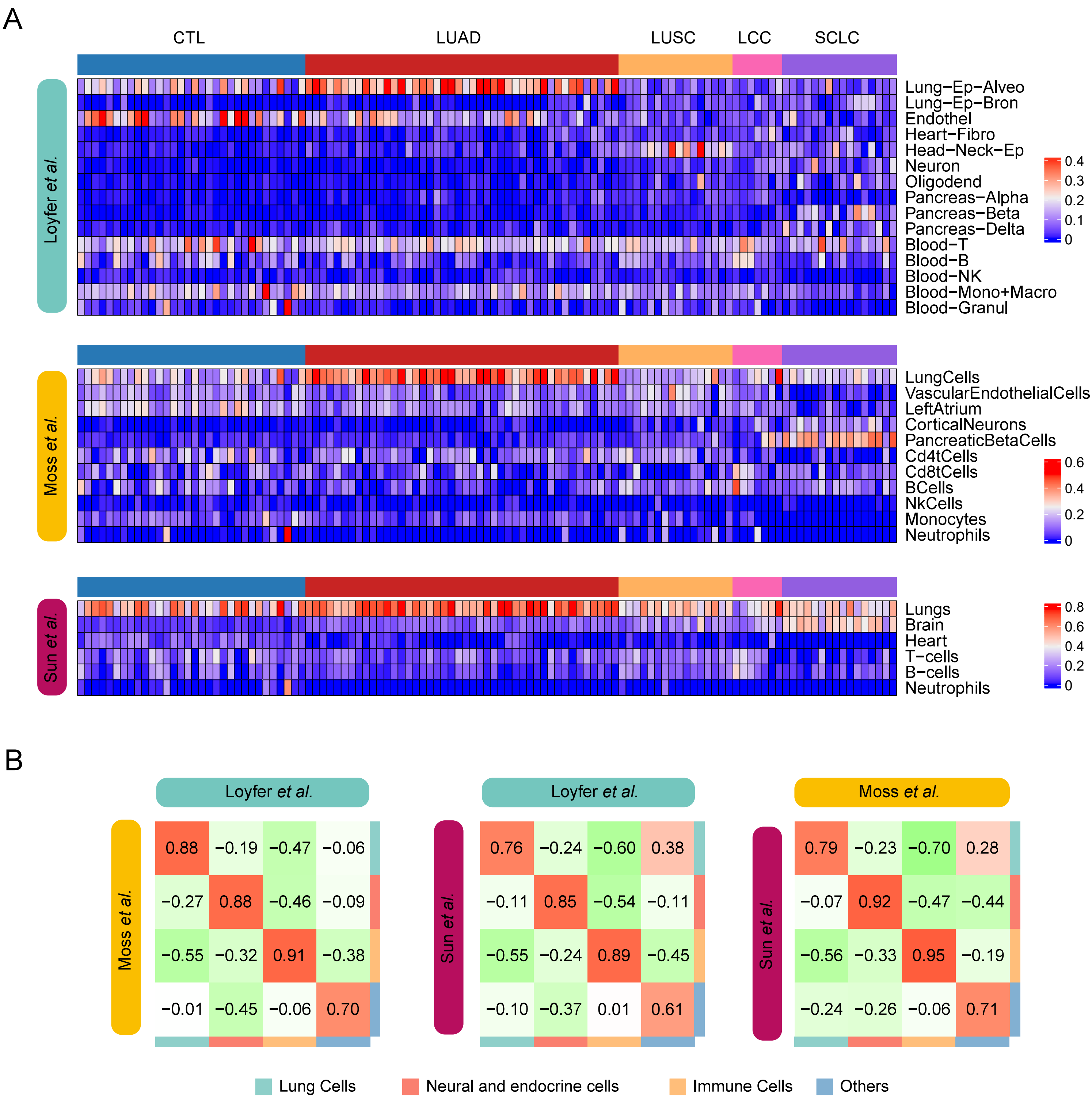
A
CTL
LUAD
LUSC
LCC
SCLC
Loyfer et al.
Lung−Ep−Alveo
Lung−Ep−Bron
Endothel
Heart−Fibro
Head−Neck−Ep
Neuron
Oligodend
Pancreas−Alpha
Pancreas−Beta
Pancreas−Delta
Blood−T
Blood−B
Blood−NK
Blood−Mono+Macro
Blood−Granul
0.4
0.3
0.2
0.1
0
Moss et al.
LungCells
VascularEndothelialCells
LeftAtrium
CorticalNeurons
PancreaticBetaCells
Cd4tCells
Cd8tCells
BCells
NkCells
Monocytes
Neutrophils
0.6
0.4
0.2
0
Sun et al.
Lungs
Brain
Heart
T−cells
B−cells
Neutrophils
0.8
0.6
0.4
0.2
0
B
Loyfer et al.
Moss et al.
0.88 −0.19 −0.47 −0.06
−0.27 0.88 −0.46 −0.09
−0.55 −0.32 0.91 −0.38
−0.01 −0.45 −0.06 0.70
Loyfer et al.
Sun et al.
0.76 −0.24 −0.60 0.38
−0.11 0.85 −0.54 −0.11
−0.55 −0.24 0.89 −0.45
−0.10 −0.37 0.01 0.61
Moss et al.
Sun et al.
0.79 −0.23 −0.70 0.28
−0.07 0.92 −0.47 −0.44
−0.56 −0.33 0.95 −0.19
−0.24 −0.26 −0.06 0.71
Lung Cells
Neural and endocrine cells
Immune Cells
Others
