# Supplementary material for: Pattools‑implemented methylation vector analysis reveals aberrant subtype‑specific methylation in lung cancer across tissue and plasma cfDNA
Source: Clin Transl Med. 2026 Aug 28;16(9):e70812. doi: 10.1002/ctm2.70812 (PMC13522992; doi:10.1002/ctm2.70812)
Supplement: Supplementary file 3 [file CTM2-16-e70812-s003.pdf]

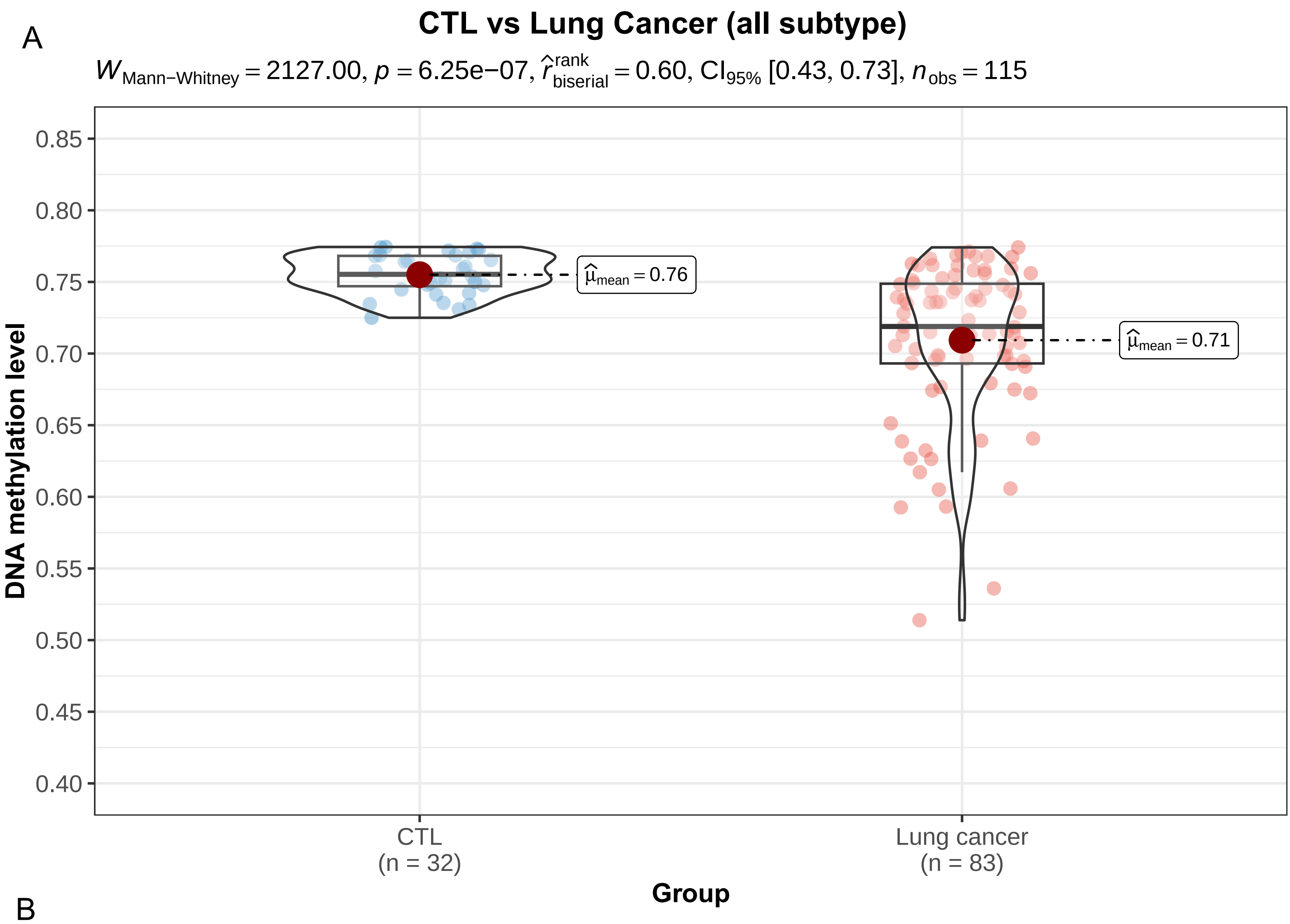
A
CTL vs Lung Cancer (all subtype)
$W_{\text{Mann-Whitney}} = 2127.00$, $p = 6.25\text{e}{-07}$, $\hat{r}^{\text{rank}}_{\text{biserial}} = 0.60$, $\text{CI}_{95\%}$ [0.43, 0.73], $n_{\text{obs}} = 115$
DNA methylation level
0.85
0.80
0.75
0.70
0.65
0.60
0.55
0.50
0.45
0.40
$\hat{\mu}_{\text{mean}} = 0.76$
$\hat{\mu}_{\text{mean}} = 0.71$
CTL
(n = 32)
Lung cancer
(n = 83)
Group

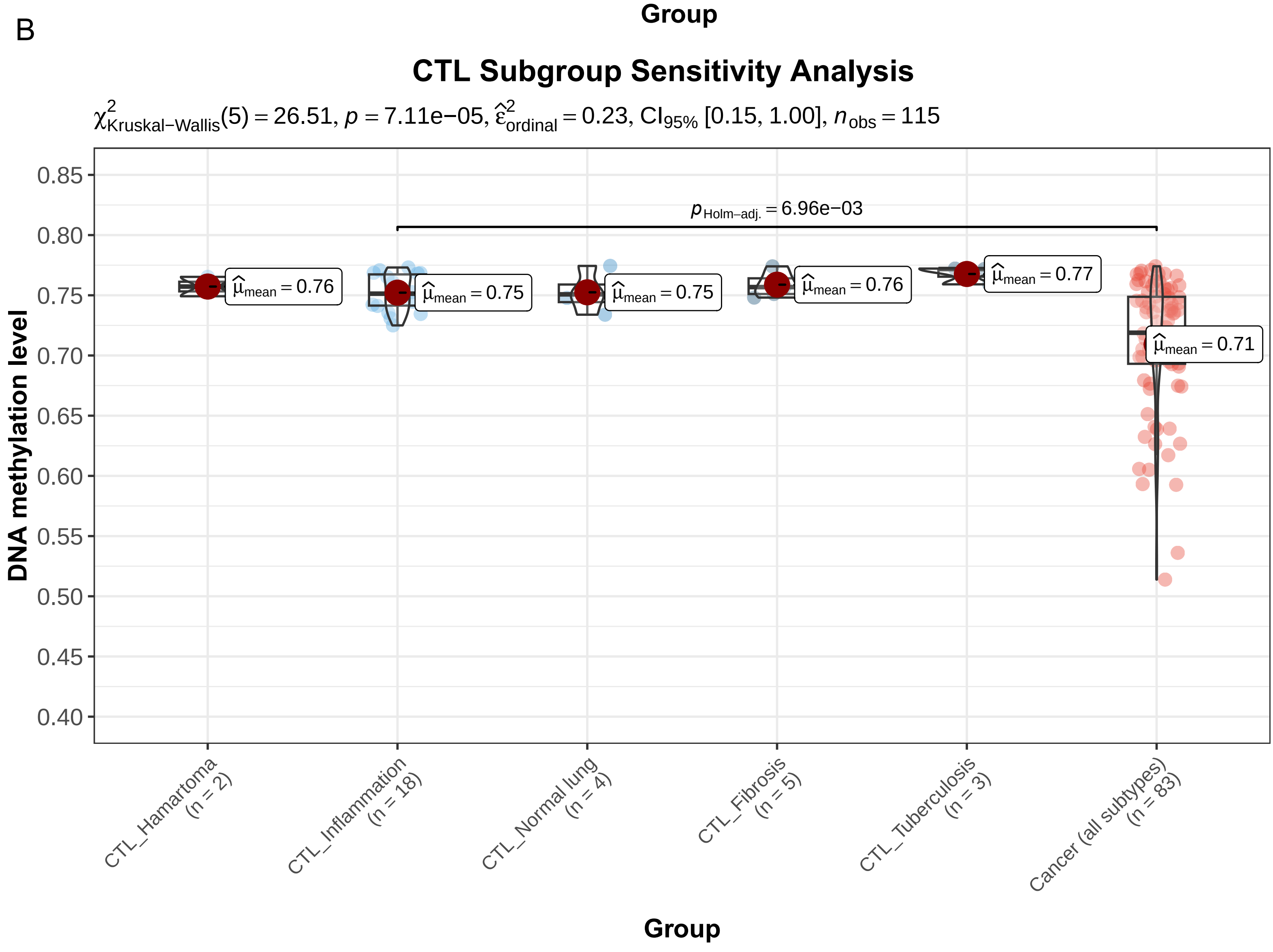
B
CTL Subgroup Sensitivity Analysis
$\chi^2_{\text{Kruskal-Wallis}}(5) = 26.51$, $p = 7.11\text{e}{-05}$, $\hat{\varepsilon}^2_{\text{ordinal}} = 0.23$, $\text{CI}_{95\%}$ [0.15, 1.00], $n_{\text{obs}} = 115$
DNA methylation level
0.85
0.80
0.75
0.70
0.65
0.60
0.55
0.50
0.45
0.40
$p_{\text{Holm-adj.}} = 6.96\text{e}{-03}$
$\hat{\mu}_{\text{mean}} = 0.76$
$\hat{\mu}_{\text{mean}} = 0.75$
$\hat{\mu}_{\text{mean}} = 0.75$
$\hat{\mu}_{\text{mean}} = 0.76$
$\hat{\mu}_{\text{mean}} = 0.77$
$\hat{\mu}_{\text{mean}} = 0.71$
CTL_Hamartoma (n = 2)
CTL_Inflammation (n = 18)
CTL_Normal lung (n = 4)
CTL_Fibrosis (n = 5)
CTL_Tuberculosis (n = 3)
Cancer (all subtypes) (n = 83)
Group
