# Supplementary material for: Pattools‑implemented methylation vector analysis reveals aberrant subtype‑specific methylation in lung cancer across tissue and plasma cfDNA
Source: Clin Transl Med. 2026 Aug 28;16(9):e70812. doi: 10.1002/ctm2.70812 (PMC13522992; doi:10.1002/ctm2.70812)
Supplement: Supplementary file 4 [file CTM2-16-e70812-s005.pdf]

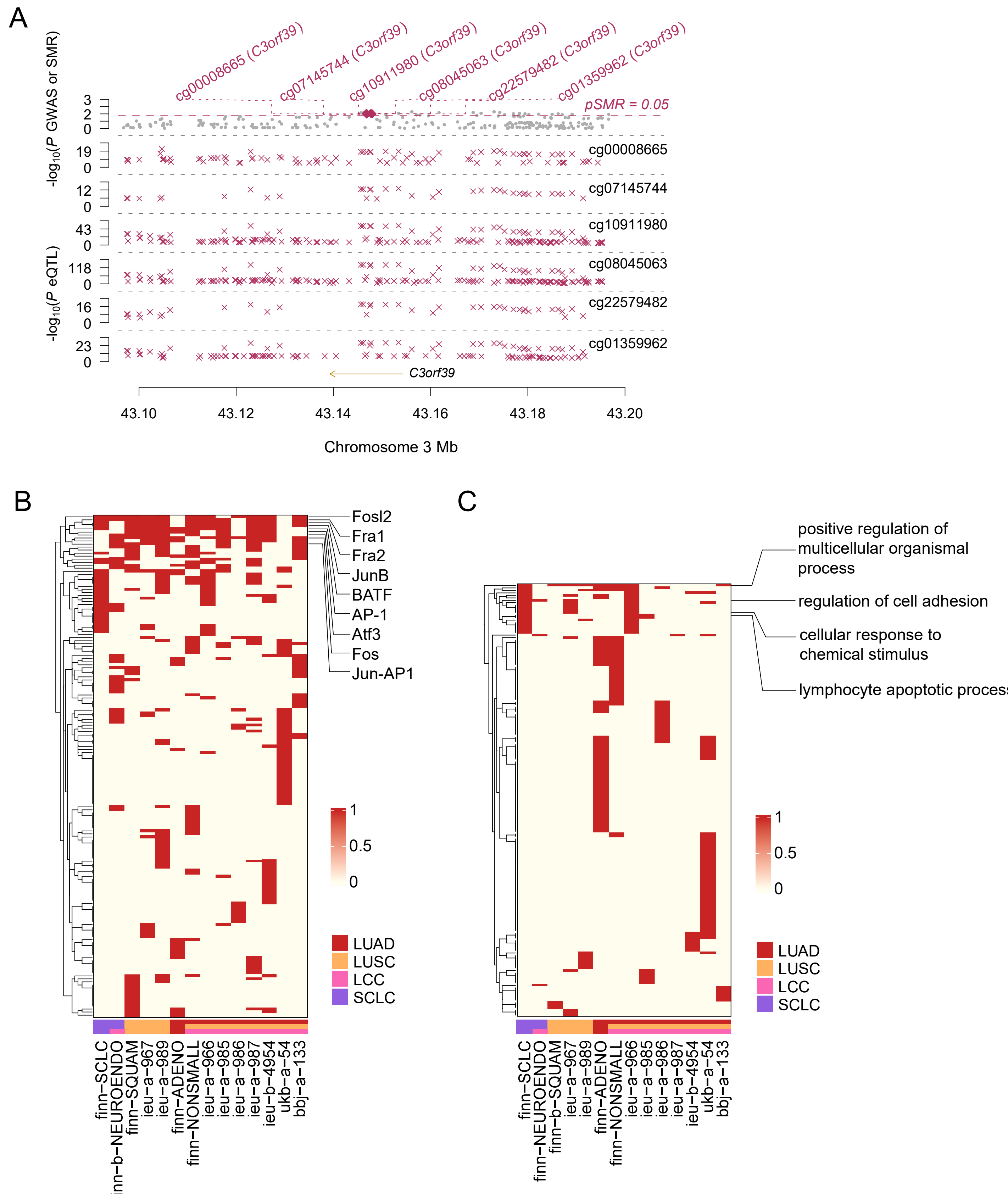
A
cg00008665 (C3orf39)
cg07145744 (C3orf39)
cg10911980 (C3orf39)
cg08045063 (C3orf39)
cg22579482 (C3orf39)
cg01359962 (C3orf39)
pSMR = 0.05
-log10(P GWAS or SMR)
-log10(P eQTL)
cg00008665
cg07145744
cg10911980
cg08045063
cg22579482
cg01359962
C3orf39
43.10
43.12
43.14
43.16
43.18
43.20
Chromosome 3 Mb
B
Fosl2
Fra1
Fra2
JunB
BATF
AP-1
Atf3
Fos
Jun-AP1
1
0.5
0
LUAD
LUSC
LCC
SCLC
finn−SCLC
finn−b−NEUROENDO
finn−SQUAM
ieu−a−967
ieu−a−989
finn−ADENO
finn−NONSMALL
ieu−a−966
ieu−a−985
ieu−a−986
ieu−a−987
ieu−b−4954
ukb−a−54
bbj−a−133
C
positive regulation of multicellular organismal process
regulation of cell adhesion
cellular response to chemical stimulus
lymphocyte apoptotic process
1
0.5
0
LUAD
LUSC
LCC
SCLC
finn−SCLC
finn−NEUROENDO
finn−b−SQUAM
ieu−a−967
ieu−a−989
finn−ADENO
finn−NONSMALL
ieu−a−966
ieu−a−985
ieu−a−986
ieu−a−987
ieu−b−4954
ukb−a−54
bbj−a−133
